# Methodological review of the design, objectives and sample size of Research for Patient Benefit (RfPB) applications that use an external randomised controlled pilot trial design: A protocol

**DOI:** 10.1371/journal.pone.0343981

**Published:** 2026-03-03

**Authors:** Claire L. Chan, Saskia Eddy, Jennie Hejdenberg, Ben Morgan, Heather M. Morgan, Gillian Lancaster, Clare Robinson, Sandra M. Eldridge

**Affiliations:** 1 Centre for Evaluation and Methods, Wolfson Institute of Population Health, Queen Mary University of London, London, United Kingdom; 2 Department of Primary Care and Public Health, Brighton and Sussex Medical School, Brighton, United Kingdom; 3 NIHR Coordinating Centre, Twickenham, United Kingdom; 4 School of Medicine, Medical Sciences and Nutrition, University of Aberdeen, Foresterhill, Aberdeen, United Kingdom; 5 School of Medicine, Keele University, Keele, United Kingdom; 6 Bristol Trials Centre, Bristol Medical School, University of Bristol, Bristol, United Kingdom; University of Benghazi, LIBYA

## Abstract

**Background:**

The National Institute for Health and Care Research accepts applications for pilot and feasibility studies to their Research for Patient Benefit (RfPB) programme. There has been limited work describing the design practices of these applications and funding status. Knowing some of the qualities which may contribute towards a pilot or feasibility study application successfully gaining funding could help researchers improve the quality of their applications. Therefore, this study describes the protocol for a review looking at the characteristics of funded and non-funded external pilot trial applications. In particular, the primary objective is to describe the planned sample size and sample size justifications.

**Methods:**

The study will be conducted on 100 applications from Competition 31–37 with a randomised feasibility design, identified and given access to us by RfPB where the lead applicant has consented. We will screen these applications to identify the external pilot trials, first looking through the titles and then the full text. Following this, we will extract data on information such as medical area, study design, objective(s), sample size, sample size justification, and funding outcome stage one and two. Validation will be performed on 20% of the data extracted; discrepancies will be resolved by discussion or a third reviewer will decide if there is no consensus. We will use descriptive statistics to summarise quantitative data, and will analyse qualitative data using thematic analysis. Findings will be summarised through discussion with the project contributors to produce a reader-friendly guidance document.

**Discussion:**

This work will provide a more complete picture of RfPB external randomised pilot and feasibility trials. The findings will assist researchers when planning their pilot trials, and could help improve the quality of submitted applications.

**Protocol Registration:**

Open Science Framework protocol registration DOI: https://doi.org/10.17605/OSF.IO/PYKVG.

## Introduction

In England the largest funder of health and care research is the NIHR [1]. In 2006 as part of an effort to consolidate health research support, the NIHR was created [2,3]. The NIHR mainly receives funding from the Department of Health and Social Care [1]. The NIHR has thirteen research programmes [4] which are available for applicants to submit applications for health care research. One of the thirteen programmes is the Research for Patient Benefit (RfPB) programme, which focuses on interventions which are *‘concerned with the day-to-day practice of health service and social care staff’* [5].

**Fig 1:**
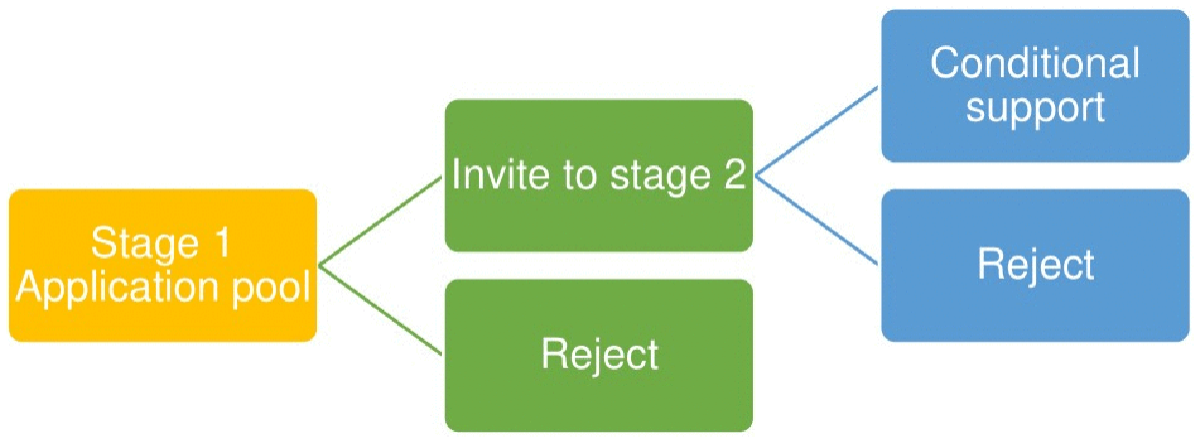
RfPB two-stage application process.

Several of the NIHR’s funding streams, including RfPB, have within their remit pilot and feasibility studies (PAFS) which focus on addressing areas of uncertainty before the main study commences [6]. Research supported by the RfPB programme must demonstrate it is clearly beneficial in terms of the health or wellbeing of users of the National Health Service (NHS) [5]. Within the RfPB programme, feasibility studies typically receive a maximum of £300,000 [7], within which costs must demonstrate a clear pathway to patient benefit. The RfPB programme has a two-stage application process; after each stage applications are reviewed by a multidisciplinary committee who decide whether to reject or support the application as shown by Fig 1.

The NIHR provide some guidance for researchers as to what constitutes a pilot study [8]. Specifically, the NIHR have adopted the definitions by Eldridge and colleagues who describe a feasibility study as a study that asks ‘*whether something can be done, should we proceed with it, and if so, how*’, and a pilot study as a study that asks the same question but also ‘*has a specific design feature: in a pilot study a future study, or part of a future study, is conducted on a smaller scale*’ [9]. Pilot studies are considered a subset of feasibility studies, and randomised pilot studies further include the randomisation of participants. External pilot studies are studies where the outcome data are not included as part of the main trial outcome data set [10]. Within this research we focus on external randomised pilot studies.

There has been limited work describing and assessing pilot and feasibility study applications submitted to RfPB, and in particular the experimental design, study objectives and sample size practices of both funded and non-funded applications. Fairhurst and colleagues [11] conducted a review of NIHR funded surgical protocols, both randomised and non-randomised, and identified a median sample size of 50 participants (range (30–200)), in randomised surgical PAFS trials funded by RfPB. However, Fairhurst and colleagues [11] only provided a broad overview of the eligible funded studies and do not describe in detail the sample size or the design of the trials, and did not consider non-funded trial protocols.

Similarly, Morgan and colleagues [12] provide an important summary of RfPB funded feasibility studies. Within the review [12] the outcome of the feasibility study, the funding received, plans to continue with a future definitive trial and where future funding will be sought are described. Morgan and colleagues [12] did not describe the methodological details of the feasibility study, such as experimental design, objectives and sample size. Furthermore, Morgan and colleagues [12] only considered funded studies and did not review non-funded studies. Therefore, a more detailed description of the experimental design, objectives and sample size of both funded and non-funded applications is warranted.

Within this present work, the characteristics of funded and non-funded external randomised controlled pilot study stage one and stage two applications will be described. At present, this methodological review of RfPB applications, alongside committee feedback, would be the first to provide information on the experimental design, objectives and planned sample size practices. This work will build upon previous research [11,13] to provide a more complete picture of RfPB randomised pilot and feasibility trials.

## Materials and methods

### Research aims

The overall research aim is to gain an insight into the sample size practices of funded and non-funded RfPB applications. Specifically we aim to:

- Describe the methodological characteristics of a sample of external randomised pilot applications that were successful, as well as unsuccessful, at obtaining RfPB funding.
- Gain a better understanding of the sample size practices used by researchers who apply for RfPB funding.
- Develop an understanding of what differs in terms of sample size practices, experimental design and objectives, between external randomised pilot trials which obtain RfPB funding and those which do not.
- Gain an insight into committee feedback provided to researchers of funded and non-funded pilot trials.
- Provide guidance to assist researchers when planning their pilot trials

### Research objectives

Our primary and secondary objectives are as follows:

### Primary objective

1. Describe the planned sample size and sample size justification of funded and non-funded applications.

### Secondary objectives

1. Describe the methodological characteristics of funded and non-funded applications focusing on:
  i. Planned primary and secondary objectives.
  ii. Experimental design.
2. Synthesize committee feedback related to sample size practices such as justifications, as well as the experimental design and objectives in funded and non-funded applications.
3. To explore whether there is an association between the objectives and the sample size justification.
4. To explore whether there is an association between the methodological characteristics and whether the application received funding. In particular we aim to answer the following questions:
  i. Are studies that state their feasibility related objective(s) successful or unsuccessful at obtaining funding?
  ii. Are studies that state their sample size justification(s) successful or unsuccessful at obtaining funding?
  iii. Is there an association between the sample size justification and whether the application received funding?
5. Describe how the planned sample size justifications change between stage one and stage two, if applicable.
6. Produce guidance based on the above findings that could be used to assist researchers when planning their pilot trials.

### Study design

The protocol was developed in line with the PRISMA-P statement [14] and elaboration and explanation document [15] (S1 Checklist). The protocol is registered on Open Science Framework, a research project management tool [16]. The registration DOI is https://doi.org/10.17605/OSF.IO/PYKVG

### Eligibility criteria

As the terms *‘pilot’* and *‘feasibility’*, as well as the terms *‘trial’* and *‘study’* are often used synonymously within the PAFS literature, they will also be used synonymously in this current work. Therefore, although the focus of this current work is external randomised controlled pilot trials, if an eligible study, which is an external pilot trial by design, is described as a *‘feasibility study’* or ‘*feasibility trial’* or *‘pilot trial’* or *‘pilot study’* then the study will be considered for inclusion.

### Inclusion criteria

1. Described as a *‘pilot study’*, *‘feasibility study’*, *‘pilot trial’*, *‘feasibility trial’.*
2. Within the context of any area of health care research.
3. Randomised controlled trial.
4. External pilot design.
5. Primary research.
6. Applications with a funding decision received from RfPB between July 2017 to July 2019 inclusive.

The dates chosen were in line with a request from other collaborators who are not involved within this present research but were similarly requesting applications from RfPB for research purposes [17].

### Exclusion criteria

1. Non pilot/feasibility experimental design.
2. Economic evaluation.
3. Internal pilot design.

RfPB only fund English organisations and do not fund pure audits or animal research. Therefore any non-English, health care audits, and non-human applications are by definition excluded.

### Information sources

Two sources of data will be used, namely NIHR RfPB stage one and two pilot and feasibility study applications, and the corresponding outcome letters. The Secretary of State for Health and Social Care is acknowledged as the source of the data, in line with the Data Sharing Agreement between the Secretary of State for Health and Social Care of 39 Victoria Street, Westminster, London, SW1H 0EU (the Provider Institution) and Queen Mary University of London (the Recipient Institution).

### Search strategy

RfPB had 918 stage one applications from Competition 31 (grant awarded in July 2017) to 37 (grant awarded in July 2019). RfPB searched the titles and plain English summaries for keywords ‘feasibility’, ‘feasible’ and ‘pilot’, and found 341 feasibility studies. They then searched the titles and plain English summaries for random* to identify those with a randomised design. Those that did not have random* in the title or plain English summary, they reviewed the research plan to identify whether the design was randomised or not. This gave 269 applications for a feasibility study with a randomised design. Of these 269 applications, 110 had been invited to stage two of the RfPB application process (41%).

The 269 applications corresponded to 236 lead applicants who were contacted by RfPB for their consent. Of these, 89 applicants consented, corresponding to 100 applications, of which 53 applications made it to stage two of the RfPB application process (53%). Chief investigator permission was sought from RfPB using the standard wording in S2 Appendix. JH and BM created the wording of consent requests to RfPB applicants in line with their legal team, and contacted researchers using the consent form to ask for their permission to share research funding applications with us (as well as a team at the University of Oxford doing a similar project). JH and BM accessed the applications between 12^th^ March 2021 and 30^th^ June 2021, and had access to identifying information as they collected and anonymised the applications. We will only be using the redacted research funding applications in this project.

We submitted a formal ethics application for review to Queen Mary, University of London ethics team. We were advised that the project did not need ethical approval and obtained a formal letter of objection for this (S3 File).

### Application selection process

We will screen the 100 applications available to us to identify those satisfying all the eligibility criteria. The full text RfPB applications will be reviewed. Any applications that fulfil the inclusion criteria and do not meet any of the exclusion criteria will be eligible. If the eligibility of any applications are unclear after reviewing the full text RfPB application then SE, GL or CR will be consulted. At most 100 eligible RfPB pilot study stage one applications will therefore be included within the review, along with any of their respective stage two applications. A precision-based sample size calculation shows that 100 applications would enable us to estimate the proportion of applications reporting a sample size justification to within approximately 8%, assuming a 95% desired confidence level and an estimated proportion of 0.80.

### Data extraction

We have created a bespoke data extraction sheet in Excel to record the data. The data extraction form was piloted by CC on 10% of eligible articles to determine if additional fields were required, or items in the codebook needed further clarification. Based on this, Table 1 shows the data that will be extracted from the RfPB applications, if available:

**Table 1:** Data extraction items.

Item	Extraction item	Value	Notes
1	Identification number	Number	
2*	Medical area	• Oncology • Respiratory/Cardiology • Gastroenterology/Hepatology • Paediatrics • Mental health • Women’s health • Oral health • Musculoskeletal/Orthopaedics • Infection • Haematology • Ophthalmology • Endocrinology • Emergency/critical care • Other	
3*	If respiratory, is it related to asthma?	• Yes • No	
4*	Study description	• Described as a pilot study • Described as a feasibility study • Use both terms	How the study is described by the applicant.
5*	Intervention description	• Drug • Surgery/Procedure • Medical device/Equipment • Education/Counselling • Physiotherapy/Exercise • Other	
6*	Experimental design: Trial design	• Parallel arm trial • Crossover trial • Factorial trial • Other (describe)	Trial design according to how participants receive the intervention.
7*	Experimental design: Unit of randomisation	• Individuals • Clusters • Other	
8*	Experimental design: Number of arms randomised to	Number	
9*	Research objective(s)	Text (categorised during analysis)	
10*	Specify any of the objective(s) as primary?	• Yes • No	
11*	If yes, primary research objective(s)	Text (categorised during analysis)	
12*	Total planned sample size	Number	If the experimental design of the trial is a cluster-randomised trial, there may be a cluster level sample size, such as the number of countries, schools, hospitals etc, as well as an individual level sample size, such as the number of patients.
13*	State sample size justification?	• Yes • No	Justification could be for a variety of reasons such as practical, statistical, financial, other, or may not be reported. We will also collect information on any methodological papers or guidelines they followed (if any) in putting the application together.
14*	If yes, sample size justification	Text (categorised during analysis)	
15*	Papers/guidelines referenced for sample size justification?	• Yes • No	
16*	If yes, papers/guidelines referenced for sample size justification	Text	
17*†	Funding committee panel outcome	• Rejected • Conditional Support • Awarded	
18*	Funding committee feedback related to sample size	Text	
19*	Funding committee feedback related to objectives	Text	
20*	Funding committee feedback related to experimental design	Text	
21	Related to another application?	• Yes • No	Some studies are resubmitted in a later competition round so are related.

* These items are extracted again for the stage 2 application, where applicable. † For item 17, the categories at stage 1 are “Rejected/Conditional Support” and the categories at stage 2 are “Awarded/Rejected”.

CC will extract data from eligible applications, with SEd verifying 20% of the data extracted. If discrepancies, *‘pairs of statements that could not both be true’* are found, then these will be resolved by discussion, referring to SE, GL or CR if there is no consensus. If any information is unclear, a note of this will be made in the review.

We expect data collection to be completed by May 2026.

### Sub study

A case study will be performed on any pilot trial applications identified related to a common medical area, asthma, as part of work funded by the Asthma UK Centre for Applied Research. Asthma is a common long-term lung condition that affects about 8 in every 100 people in the UK, with four people estimated to die every day in the UK from asthma attacks [18]. It is therefore an important health topic and there is a need to make sure that research related to asthma is completed to a high standard [19]. Based on sifting, we expect around 5 applications to be specifically related to asthma. Therefore, we will focus on descriptive summaries. In particular, for these applications, we will describe the planned sample size and sample size justification of the funded and non-funded applications, and summarise any sample size related committee feedback. This will provide the opportunity to explore in more depth sample size practices of RfPB applications related to asthma.

### Analysis

A record of the number of articles assessed at each stage in the study selection process will be made and a PRISMA flow chart will be produced [20].

The primary outcomes will be the planned sample size and the planned sample size justification(s). Secondary outcomes will be the planned primary objective(s), the planned secondary objective(s), the experimental design, and the sample size related committee feedback contained within the outcome letters.

### Quantitative analysis

Variables that appear to follow a continuous normal distribution will be summarised with means and standard deviations; otherwise, medians, interquartile ranges and ranges will be used. We will summarise categorical variables using frequencies and percentages. All summarised data will be presented in tables, and in particular for the objectives looking at associations, we will present two-way tables. We will not perform statistical tests when looking at associations as our study is not powered to do so. All analyses will be conducted using the statistical software Stata version 18.5 or higher [21].

### Qualitative analysis

A pragmatic paradigm will be utilised for this research [22]. The outcome letters will be analysed by SEd using thematic analysis [23], based on the results of a piloting exercise of a random 10% sample of the outcome letters. Thematic analysis will broadly follow the following steps suggested by Braun and Clarke [23]: familiarisation of the data, generating codes, identifying themes or categories, reviewing themes or categories, defining and naming themes or categories, and identifying examples from the data. We will not monitor for data saturation or information power, as there is no possibility of modifying the analysis sample beyond the 100 applications that have been made available to us.

An inductive approach will be used whereby codes and categories will be identified, defined and labelled in response to the data as SEd reads the outcomes letters and uses her interpretation. An inductive approach involves identifying codes as we read through the data. An inductive approach is routinely used when exploring new phenomena [24], which is appropriate here as we explore previously unexamined committee feedback, and there is a lack of clarity as to whether there are stock phrases or structured feedback or open text in the committee feedback. Furthermore, the variety of different RfPB funding committees, their varying personnel and the range of years of the committee feedback letters means that an inductive approach is the most appropriate approach in the first instance. Reflective memos will be recorded, which will contain SEd’s and HMM’s personal impressions whist reading the committee feedback and a description of how and why key analytical decisions were made. The reflective memos will act as an audit trail. SEd and HMM will practice reflexivity throughout this research. HMM will independently double code the codes and categories from at least 10% of outcome letters. Any major discrepancies regarding the codes and categories will be discussed by SEd and HMM until consensus. If consensus cannot be reached SE, GL or CR will be consulted and an additional 10% will be double coded. Qualitative data will be managed and digitally coded within Microsoft Word and Excel. After the coding frame has been finalised, illustrative examples of each code will be identified from the outcome letters and will be reported. The results from this research will be reported in line with the Standards for Reporting Qualitative Research (SRQR) guidelines [25]. While conducting our analysis, we will present our research at institutional work in progress meetings for feedback to identify and address possible biases.

Findings from all the objectives, quantitative and qualitative, will be summarised through discussion with the project contributors to produce a reader-friendly guidance document to assist researchers when planning their external pilot trials.

## Discussion

There has been limited work summarising the experimental design, study objectives and sample size practices of both funded and non-funded pilot and feasibility study applications. This work will provide a more complete picture of RfPB external randomised pilot and feasibility trials.

We acknowledge some potential limitations of our study. Firstly, our data source is applications from 2017 to 2019, which may not accurately reflect the current situation in a continuously evolving field of medical research. However, the field of sample size in pilot studies has not changed materially between 2019 and now, and in particular there has been no definitive guidance about sample size in pilot trials published since 2019. Therefore, we feel that the dates of the data source are acceptable, but we would suggest that this study should be replicated after significant guidance emerges on sample size in pilot trials.

Another potential limitation of our study is that we are limited to the redacted research plan of the funding applications, and so it is possible that we might miss out on some information if it has only been reported elsewhere in the application. We anticipate that this will be unlikely though and expect that the data we are interested in should mostly be available from the application sections we have.

Obtaining consent to include applications in our study may have introduced bias. Of the 269 applications for a feasibility study with a randomised design identified by RfPB, 41% were invited to stage two of the RfPB application process. However of the 100 consented applications, 53% made it to stage two of the RfPB application process. This suggests that researchers who were awarded funding were perhaps more likely to consent than those who were not.

Finally, we acknowledge that some of our extractions may be subjective, in particular the qualitative assessment of the funding committee feedback. However, a systematic approach will be used and pre-defined methods as much as possible, as detailed in this protocol. The themes generated from our qualitative analysis could be used as a framework for deductive analysis in future.

The findings from this study could help healthcare researchers improve the quality of their submitted applications. In turn this could result in researchers gaining funding quicker and reduce the length of time from discovering a potentially beneficial intervention to the intervention being utilised by patients, something that the NIHR are keen on as demonstrated by their *‘Push the Pace’* project [26]. Moreover, an increase in higher quality applications would also likely reduce the length of time committee members and reviewers spend reviewing applications that are unlikely to receive funding, which has been noted as a particularly onerous task [27]. This protocol will assist in carrying out our methodological review by ensuring transparency in the methods adopted, and allowing any future updates of this review to be reproduced.

## Supporting information

S1 Checklist PRISMA-P 2015 checklist. (DOCX)

S2 Appendix Wording used by RfPB to obtain consent. (DOCX)

S3 File Letter from QM ethics. (PDF)
