# Supplementary material for: Methodological review of the design, objectives and sample size of Research for Patient Benefit (RfPB) applications that use an external randomised controlled pilot trial design: A protocol
Source: PLoS One. 2026 Mar 3;21(3):e0343981. doi: 10.1371/journal.pone.0343981 (PMC12956074; doi:10.1371/journal.pone.0343981)
Supplement: S2 Appendix — (DOCX) [file pone.0343981.s002.docx]

S2 Appendix. Wording used by RfPB to obtain consent.

**Subject: Request to use information from RfPB application in support of further research** [[Reference]]

Dear [[Lead Applicant name]],

RE: [[Reference]] – [[Title]]

The NIHR Research for Patient Benefit programme is conducting research on submitted funding applications for randomised pilot and feasibility trials in collaboration with third party UK Higher Education Institutions. The purpose of the research is to review the methodological characteristics of funding applications for pilot and feasibility trials, and to assess funding outcomes (Stage 1 and Stage 2) to help improve the design of pilot and feasibility studies.

You are being approached for permission as you were the lead applicant of a RfPB application. We would like your permission to use some of the information from your application form as part of this research and to share the following information with our collaborating third-party UK Higher Education Institutions.

**Proposed Research Use**

The information we are proposing to use to support research is:

- Parts of the application form (the research plan only from Stage 1 and Stage 2 (if applicable) – all personal details will be redacted)
- The outcome letters (Stage 2 and/or Stage 1) containing committee feedback (all personal details will be redacted from the letters).

The information will be sent to the relevant HEI via encrypted transfer.

**Permission**

We need your permission to use the information in your RfPB application to support this research. Our guidance notes for applicants make it clear that the application will be treated as confidential and acknowledge that the text in application belongs to the applicant and the submitting institution by copyright.

Please reply to this email, copying and pasting the three points below and confirming each of the points by stating YES or No to each point.

**Please ensure that you consult with any relevant parties (such as those with signing authority within your institution and/or any co-applicants whose information may appear in the application form) before you respond.**

**Sharing the application form**

- Please confirm, on behalf of the institution that submitted the application, that RfPB may use the text including any confidential information contained in the application only for the proposed Research Use outlined above. No personal information will be shared. **YES/NO**

- Please confirm, on behalf of the institution that submitted the application, that RfPB may use and share the text, data or images used in the application only for the proposed Research Use outlined above.  **YES/NO**

- Please confirm that RfPB may use and share the text, data or images used in the application that is owned or controlled by a co-applicant or other third party only for the proposed Research Use outlined above. **YES/NO**

We hope that you will support our endeavours to improve the design of pilot and feasibility studies through this research and would be very grateful for your permission to include your application.

If you have any questions or need clarification regarding this request, please contact me.

Kind regards

The RfPB team/NAME
