# Supplementary material for: Methodological review of the design, objectives and sample size of Research for Patient Benefit (RfPB) applications that use an external randomised controlled pilot trial design: A protocol
Source: PLoS One. 2026 Mar 3;21(3):e0343981. doi: 10.1371/journal.pone.0343981 (PMC12956074; doi:10.1371/journal.pone.0343981)
Supplement: S3 File — (PDF) [file pone.0343981.s003.pdf]

Queen Mary University of London
Joint Research Management Office
Dept. W
69-89 Mile End Road
London E1 4UJ
www.jrmo.org.uk
research-ethics@qmul.ac.uk
Tel: 020 7882 7915 / 6947

15 November 2024

Cc: Ms Saskia Eddy, Dr Clare Robinson

Dear Mrs Chan

Reference number: QME24.0716
Study title – Methodological review of the design, objectives and sample size of Research for Patient Benefit (RfPB) applications that use an external randomised controlled pilot trial design

Having received a Research Ethics Application for the study detailed above, it has been screened by the Research Ethics Facilitator(s) and a decision that no further ethical review is required. This was based on the following information:

- The study involves no human data

Extensions and Amendments:
Amendments to the research after approval or extensions to the study beyond the expiry date must be submitted via 'Create Sub Form' in the QMEthics system.

In the event of any problems or queries, do not hesitate to contact the Research Ethics Facilitators: research-ethics@qmul.ac.uk

Signed: Dr Claire-Elise Willems

Research Ethics Facilitator, on behalf of the Queen Mary Ethics of Research Committee
